# Increasing transcultural competence in clinical psychologists through a web-based training: study protocol for a randomized controlled trial

**DOI:** 10.1186/s13063-023-07878-w

**Published:** 2024-01-20

**Authors:** Selina Studer, Maria Kleinstäuber, Ulrike von Lersner, Cornelia Weise

**Affiliations:** 1 Department of Psychology, Division of Clinical Psychology and Psychotherapy, Philipps-University Marburg, Marburg, Germany; 2 Department of Psychology, Emma Eccles Jones College of Education and Human Services, Utah State University, 6405 Old Main Hill, Logan, UT 84321 USA; 3 Private practice for psychotherapy, Berlin, Germany

**Keywords:** Web-based training program, Transcultural competence, Culture sensitive psychotherapy, RCT

## Abstract

**Background:**

In mental health care, the number of patients with diverse cultural backgrounds is growing. Nevertheless, evaluated training programs for transcultural competence are missing. Barriers for engaging in transcultural therapy can be identified in patients as well as in therapists. Besides language barriers, clinical psychologists report insecurities, for example, fear of additional expenses when involving a language mediator, ethical concerns such as power imbalances, or fear of lack of knowledge or incorrect handling when working with patients from other cultures. Divergent values and concepts of disease, prejudices, and stereotyping are also among the issues discussed as barriers to optimal psychotherapy care. The planned study aims to empower clinical psychologists to handle both their own as well as patients’ barriers through a web-based training on transcultural competence.

**Methods:**

The training includes 6 modules, which are unlocked weekly. A total of *N* = 174 clinical psychologists are randomly assigned to two groups: the training group (TG) works through the complete training over 6 weeks, which includes a variety of practical exercises and self-reflections. In addition, participants receive weekly written feedback from a trained psychologist. The waitlist control group (WL) completes the training after the end of the waiting period (2 months after the end of the TG’s training). The primary outcome is transcultural competence. Secondary outcomes consist of experiences in treating people from other cultures (number of patients, satisfaction and experience of competence in treatment, etc.). Data will be collected before and after the training as well as 2 and 6 months after the end of the training.

**Discussion:**

This randomized controlled trial tests the efficacy of and satisfaction with a web-based training on transcultural competence for German-speaking clinical psychologists. If validated successfully, the training can represent a time- and place-flexible training opportunity that could be integrated into the continuing education of clinical psychologists in the long term.

**Trial registration:**

DRKS00031105. Registered on 21 February 2023.

**Supplementary Information:**

The online version contains supplementary material available at 10.1186/s13063-023-07878-w.

## Administrative information

Note: the numbers in curly brackets in this protocol refer to SPIRIT checklist item numbers. The order of the items has been modified to group similar items (see http://www.equator-network.org/reporting-guidelines/spirit-2013-statement-defining-standard-protocol-items-for-clinical-trials/).

**Table:** 

Title {1}	Increasing transcultural competence in clinical psychologists through a web-based training program: study protocol for a randomized controlled trial
Trial registration {2a and 2b}.	DRKS00031105, registered on February 21, 2023.
Protocol version {3}	First version (dated 04/07/2023)
Funding {4}	The researchers of the study are employed at Phillips-University Marburg or at the Utah State University through budgetary funds.
Author details {5a}	^1^ Dept. of Psychology, Division of Clinical Psychology and Psychotherapy, Philipps-University Marburg, Marburg, Germany ^2^ Dept. of Psychology, Utah State University, Logan, USA ^3^ Private practice for psychotherapy, Berlin, Germany
Name and contact information for the trial sponsor {5b}	Philipps-University Marburg Dept. of Psychology Clinical Psychology and Psychotherapy Gutenbergstrasse 18 35032 Marburg
Role of sponsor {5c}	The funder has no role in study design, analysis, interpretation, or publication of the study protocol and trial results.

## Introduction

### Background and rationale {6a}

In clinical psychological practice, we see rising numbers of patients with diverse cultural backgrounds^1^. This includes both patients living in the host country for a long time and refugees and asylum seekers who have only recently arrived in the host country [1, 2]. At the end of 2021, 89.3 million people worldwide were forced to displacement of which 1.3 million were hosted in Germany [3]. More than a quarter of residents in Germany (27.3%) had a migration background in 2021 [2]. Due to the war in Ukraine, the number of Ukrainians in Germany increased almost seven times from February to November 2022 [4]. As a result, society is becoming increasingly diverse and the health care system is facing a broad cultural diversity. It is assumed that the need for therapeutic care among incoming patients with diverse cultural backgrounds is at least as high as in the German population [5]. Studies point to an increased prevalence of mental disorders in the third generation of immigrants [6]. Higher rates of mental disorders are particularly found after forced migration (e.g., due to war or other traumatic experiences) as well as in migrants who have only recently arrived in the host country [7]. Yet patients from other cultures are underrepresented in psychotherapeutic care [8]. Several studies have shown that patients with diverse cultural backgrounds are less likely to utilize psychotherapeutic services [9–12].

Numerous reasons were postulated for this, such as limited communication skills, insufficient information about health care, and shame and fear of stigmatization [10, 13]. Mainly due to the cultural mismatch between patients and clinical psychologists, patients with diverse cultural backgrounds drop out of therapy more frequently than people without a migration background [13, 14] and treatment success is lower [15, 16].

Barriers on patients’ and therapists’ ends as well as institutional barriers can eventually explain why therapy is used less and appears to be less successful for patients with diverse cultural backgrounds [13, 15–17]. Institutional barriers include, for example, the limited availability of language mediators, a lack of funding for interpreting services, and in some countries a general lack or limited funding for psychotherapy [9, 12, 18–20]. On the patient side, language barriers, lack of knowledge about care services, and fear of stigmatization may prevent treatment seeking [21–23]. The fear of stigmatization can be accompanied by social withdrawal and isolation, which further exacerbates mental disorders [19]. On the therapist’s side, insecurities in the contact with patients from other cultures, fears of increased effort (e.g., costs for interpreters), but also stereotyping and prejudices can cause only a few patients from other cultures to actually receive treatment [13, 17, 24].

In addition to the personal suffering, economic (workplace costs, lower productivity, unemployment) and health costs are at stake [25]. The challenges presented can only be addressed through holistic solutions that target all three levels—patients, therapists, and institutional barriers.

At the institutional level, it is necessary to establish multicultural teams, psychologically trained interpreters are available, and information is provided to patients in their native language [15]. Healthcare workers should further be trained in transcultural competence and the hiring of native speakers should be promoted. Also, a reduction of access barriers, for example, through simplified language and fewer administrative barriers, is to be encouraged [13, 26].

At the patient level, potential barriers should be reduced through psychoeducational content by providing easy to understand psychoeducation on mental health and treatment options via different and readily available channels (e.g., online information platforms, local institutions, social workers, social media). Information should consist of what mental symptoms and disorders are, what a psychological treatment consists of, and where and how treatment is possible. Providing such information has been shown effective in reducing fears, uncertainties, and stigmas associated with mental health care [27, 28]. An example of such a low-threshold intervention is the so-called tea garden, an intervention for asylum seekers in which specific mental health knowledge and information on treatment options is provided [29, 30].

On the therapist level, training in transcultural therapy should be encouraged. Even though patients with diverse cultural backgrounds do not need a *different* psychotherapy, methods should be applied in a culturally sensitive way [31]. That is, therapists should be aware of patients’ potentially discrepant understanding of Western psychotherapy and migration-specific and cultural factors should be taken into account in treatment, for example when setting therapy goals, discussing explanatory models, or choosing therapeutic techniques [16, 31, 32]. Training to promote transcultural competence is therefore central to increase the therapeutic success of treatment [31, 33]. Studies showed that culturally adapted psychotherapy resulted in superior outcomes for patients with diverse cultural backgrounds [33].

For the definition of transcultural competence, the study draws on the established definition by Sue et al. [34, 35]. It emphasizes that transcultural competence is built on three pillars: *knowledge* (cognitive aspects), *awareness* of attitudes and beliefs of other cultures (affective aspects), and *skills* (behavioral aspects).

To date, there is a lack of evaluated training programs teaching and thereby increasing transcultural competence in clinical psychologists [17, 36]. This is the case even though guidelines exist that highlight learning objectives and structural and content requirements of such training [37, 38]. One training that builds upon these guidelines was recently investigated and its effectiveness was demonstrated in a pilot study [22]. As expected, the training increased the transcultural competence in clinical psychologists in Germany. In addition to transcultural competence, the therapeutic relationship was further improved. Therefore, the researchers assume that training to promote transcultural competence is not only beneficial for therapies with patients with diverse cultural backgrounds, but also has positive effects on therapies regardless of cultural background.

However, such validated training remains scarce. The COVID-19 pandemic has further restricted access to continuing education [39]. Thus, there is an increasing demand to offer training opportunities that are accessible from any location and are time-flexible, allowing participants to learn at their own pace [39]. A web-based training program could reach a wider target group. Preliminary evidence from the US suggests that a web-based training could increase transcultural competence in a pediatric context via a program consisting of six online modules [40].

For this reason, we designed and implemented an online training to promote transcultural competence among clinical psychologists. The intervention builds on the training program developed by von Lersner with contents from her book on transcultural psychotherapy [41]. A preliminary version of the program, which was primarily based on a web-based textbook approach, was tested in a pilot trial [42]. In the meantime, the training has been incorporated into an interactive web-based platform in which written information is complemented by image-, audio- and video-guided content. The multisensory learning enables the contents to be memorized more effectively [43]. Each module has a pre-designed structure that can be personalized according to the interest of the participants by displaying or skipping further information as desired.

It is expected that our study will increase transcultural competence in all three areas of transcultural competence [34, 35]: knowledge, skills, and awareness (primary outcomes). In addition, we expect that the training will increase treatment satisfaction and competence experienced when working with patients with diverse cultural backgrounds, as well as the number of patients with diverse cultural backgrounds treated (secondary outcomes). In the long term, the training is intended to be integrated into the continuing education of clinical psychologists.

### Objectives {7}

The research aims to achieve the following objectives:

- To test the efficacy of an online training to improve transcultural competence (primary outcomes) and the experience in treating patients with diverse cultural backgrounds (secondary outcomes). Transcultural competence is measured through the MCI [44] and the OnTracc-questionnaire [45]. The experience in treating patients with diverse cultural backgrounds is measured through questions asking the number of patients, the satisfaction, and the experience of competence in treatment.
- To identify possible mediators and moderators at the clinical psychologist’s level (own migration background, multilingualism, volunteering for refugees, and patients with diverse cultural backgrounds) which might influence the efficacy of the training on the primary and secondary outcomes.
- To foster easily accessible, time- and place-independent, high-quality continuing education for clinical psychologists.

### Trial design {8}

Our study is a two-armed, randomized-controlled trial (RCT) with two parallel groups (training group vs. waitlist control group) of equal size (1:1 ratio at randomization). The study is designed as a superiority trial investigating the efficacy of a 6-week transcultural competence training to improve transcultural competence in clinical psychologists as compared to a waitlist control group. Furthermore, potential mediators and moderators of the training effect will be investigated in the trial.

## Methods: participants, interventions, and outcomes

### Study setting {9}

Licensed clinical psychologists and psychologists in training (for at least one year) from German-speaking countries (Germany, Switzerland, Austria) will be recruited. As the study is web-based, participants will take part in the study online via an electronic device (tablet, PC).

### Eligibility criteria {10}

The following inclusion criteria apply to participants:

1. Licensed as clinical psychologists or ongoing clinical psychology training for at least 1 year in one of the psychotherapy procedures approved by the licensing board in the respective country
2. Currently working as clinical psychologists or psychologists in training (to be able to conduct practical exercises)
3. Access to a web-enabled device (PC, laptop) with stable Internet connection
4. Sufficiently good knowledge of German to work with the training material
5. Sufficient time and motivation to work on the training for about 1–2 h per week for 6 weeks

6. Besides the inclusion criteria, no exclusion criteria are applicable

### Who will take informed consent? {26a}

Potential trial participants receive detailed written information about the study aims and procedure, the randomization process, the content of the training, and the planned assessments. The study team can be contacted at any time by email or phone for questions and clarifications. Participants must agree to the informed consent form prior to study entry. Only after consenting, participants will be forwarded to the initial study questionnaire.

### Additional consent provisions for collection and use of participant data and biological specimens {26b}

n/a. No biological specimens are collected.

### Interventions

#### Explanation for the choice of comparators {6b}

The comparator will be a waitlist control group. To date, there is no similar training that could have been used as an active comparator. In a pilot study, a third active control group was included, which only did the theoretical part of the exercises, but not the self-reflection exercises (awareness component) [42]. Since the training has now been implemented in an interactive Internet platform, it did not seem reasonable to create a parallel training where only theoretical content would be taught. For this reason, the waitlist control condition seemed the most appropriate.

#### Intervention description {11a}

The entire web-based training was tested in a feasibility study with nine clinical psychology master’s students. In interviews, they provided feedback about the structure, content, usability, comprehensibility, and responsiveness of the platform. Criticism and suggestions for improvement were taken into account and directly implemented.

##### Format

The online training lasts 6 weeks. Every week a new module is unlocked, which is expected to take between 1 and 2 h for the participants to complete. Each module contains between 9 and 10 web pages. Modules can be interrupted at any time and continued later, but it is helpful to divide a module into no more than two blocks per week. It has proven helpful to allow 2–3 days between the completion of one module and the processing of the following module. Research indicates that spacing learning units enhances retention [46]. The training includes a symbol that appears twice per module to remind participants to take a short break.

##### Content

Each module starts with the presentation of the three most important learning objectives for this module and offers an overview of its content. Each of the six modules has a distinct focus: (1) cultural concepts, (2) cultural imprinting, (3) prejudices and discrimination, (4) migration process, (5) transcultural diagnostics, and (6) language and communication (Table 1). Each module ends with a short module summary and some concluding exercises. At the end of the module, a preview of the next module is provided. The contents of the training were built on the textbook by von Lersner and Kizilhan [41] and a preliminary version of the program was tested in a pilot trial [42]. Various experts in the field of transcultural psychotherapy collaborated on the development of the training.

**Table 1:** Content of the different modules

Week	Module name and content
1	**Culture as the salt in the soup—introduction to transcultural psychotherapy** Defining culture, culture as spheres [47], the modern concept of culture: web of significance and hybridity [48], cultural configurations of the self (egocentric, sociocentric, ecocentric, and cosmocentric) [49], the introduction of the term “transcultural competence” with its three components: knowledge, skills and awareness [34], goal setting
2	**The own vs. the other cultures—the meaning of cultural imprinting** Norms and rules in the family of origin, “cultural emancipation” [50], importance of one’s own cultural imprint (what is typically German?), raising awareness for other cultures, individualistic and collectivistic cultures, “culture traps,” universality and diversity [50]
3	**They are all the same!—On the role of prejudice & discrimination** Healthcare conditions of patients with diverse cultural backgrounds in psychotherapy [15], definition and distinction of stereotypes and prejudices, development of prejudices (categorization, stereotyping, judgment) [51], functions of prejudice (orientation, adjustment, enhancement of the collective self-esteem, defense function) [52], influence of stereotypes and prejudices on the therapeutic practice, social categorization on intergroup behavior [53], social identity theory [53], ethnocentrism [54]
4	**Of Leaving and Arriving—The Migration Process & Its Consequences** Migration stages (preparatory stage, act of migration, period of overcompensation, period of decompensation, and transgenerational phenomena) with corresponding stress and resilience factors [55], from the country of origin to German health care, the asylum procedure in Germany [56], challenges as a refugee in health care
5	**Culturally sensitive exploration—What should be considered in transcultural diagnostics?** Cultural case history, cultural concepts of distress [57], disorder-specific and culturally sensitive assessment tools, Cultural Formulation Interview [57], linguistic and cultural equivalence in translations, possible biasing influences on the diagnostic process (halo effect, singularity, culturalization, shame, simulation, etc.) [41]
6	**Understanding and being understood—therapeutic relationship, language & communication** Intercultural communication (power asymmetries, collective experiences, images of others, cultural differences) [58], cultural dimensions (high- and low-context culture) [59], language mediation in therapy (procedure, acquisition, and financing) [60], conclusion

##### Didactics

The content is taught through multisensory learning. Besides written information, images, audios, and videos serve to enhance the knowledge transfer. Practical exercises and short knowledge checks motivate the participants to implement their knowledge in practice.

All six modules address the different transcultural competencies (knowledge, skills, and awareness) equally. In order to target the knowledge component, information is provided on various important topics (e.g., the migration process, the origin and function of stereotypes, different cultural dimensions, etc.). The skills are developed in a practice-oriented manner. For example, working with a language mediator is not only explained in writing, but supplemented by a demonstration video. The awareness component is addressed through numerous self-reflection exercises to continually reflect on one’s own therapeutic practice. For example, in module 2 on cultural imprinting, participants are asked about the norms and rules in their family of origin (communication styles, time management, rituals). In module 4 on the migration process, participants are invited to reflect on their own experiences, possible “culture shocks” and how they have dealt with them. The content is always supplemented by “implications for therapy,” which give concrete indications for use in clinical practice.

For a deeper examination of the content, additional texts and information are offered, which are displayed if needed. Three fictional therapists, based on real individuals, guide participants through the training. Via the fictional characters, examples, possible answers to self-reflection questions and other exercises, and inspiration and motivation for the participants are provided.

After each module, participants receive written feedback from a supervisor about the completed exercises and upcoming questions. The supervisors are at least bachelor-level clinical psychologists and receive regular supervision from an experienced clinical psychologist. The feedback is composed of prefabricated text templates and individualized to the participants’ responses. In the feedback, participants are complimented on their engagement; then, they receive individual feedback on the exercises they have completed. If they have noted specific questions in the module, these are also answered by the supervisor. The supervisor further encourages participants to keep up their active participation. Through sending the feedback, the next module will be unlocked. Participants are accompanied throughout the training and have the opportunity to contact the supervisors.

#### Criteria for discontinuing or modifying allocated interventions {11b}

Due to the non-clinical sample, no criteria have been set to discontinue or modify the training. Participants can stop working with the training at any time, without providing an explanation There are no disadvantages from withdrawal, except that in the case of discontinuation, the participants cannot receive the certificate of attendance (i.e., continuing education credits).

#### Strategies to improve adherence to interventions {11c}

Participants are encouraged to attend the training on a weekly basis. At the end of each module, the program suggests participants to plan a timeframe to complete the next module within a week. An online calendar supports this intention. Adherence is further supported by the feedback of a supervisor, who communicates with the participants at least once a week.

If the module has not been worked on after 5 days, the supervisor sends a reminder to encourage the participant to continue. The participant is also asked if and how the supervisor can support them to continue the training. If the participant still has not continued the training 1 week later, an additional reminder email follows. If neither the training is worked on nor are the emails answered for 3 weeks, the participant receives a last e-mail where he or she is asked whether there is still interest in participation. The e-mail also contains the information that the participation will be deleted if neither there is a reply to the e-mail nor the module is processed within the next 7 days. The participant is then considered to have dropped out of the study.

#### Relevant concomitant care permitted or prohibited during the trial {11d}

n/a: A non-clinical sample is studied and no clinical intervention is provided. Previously completed training in transcultural psychotherapy will be collected as a control variable.

#### Provisions for post-trial care {30}

n/a: There is no anticipated harm and compensation for trial participation apart from a certificate of attendance. For this reason, there will be no need to provide post-trial care. Nevertheless, participants can reach out to the study management via email or telephone at any time if needed.

### Outcomes {12}

#### Primary outcome measures

**Fig. 1:**
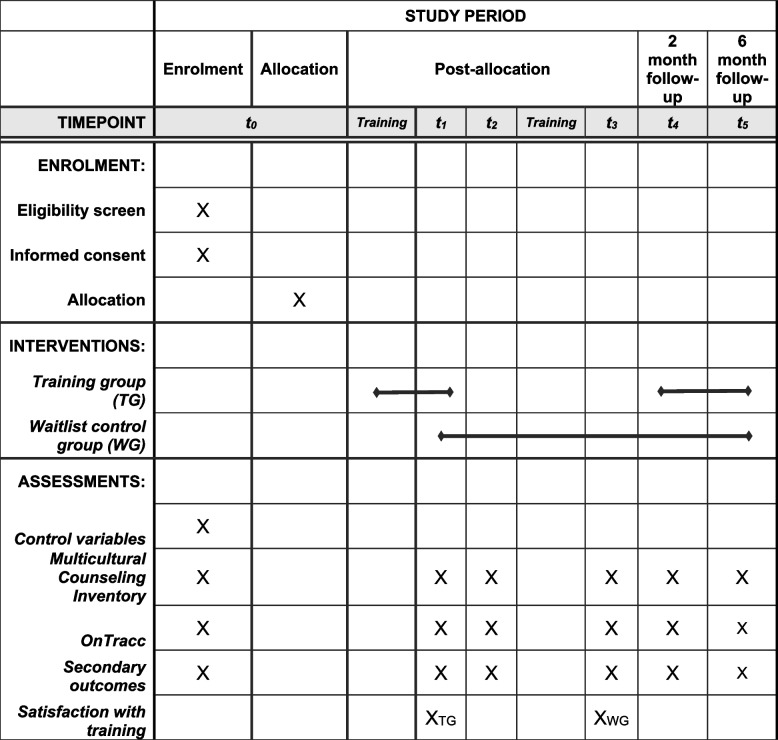
Schedule of enrolment, interventions, and assessments

The primary outcome is transcultural competence. Transcultural competence will be assessed at the beginning of the training, at the end of the training, and at the 2- and 6-month follow-up (see Fig. 1). For this purpose, the well-validated Multicultural Counseling Inventory (MCI) [44], translated into German by von Lersner [22], is used. The MCI captures general transcultural competence as conceptualized by Sue et al. [34]. With a total of 40 items, transcultural competence is assessed on the subscales *skills* (11 items, e.g., “I am able to quickly recognize and recover from cultural mistakes or misunderstandings.”), *cultural self-awareness and other-awareness* (10 items, e.g., “In order to be able to work with minority clients, I frequently seek consultation with multicultural experts and attend multicultural workshops or training sessions.”), and knowledge (11 items, e.g., “I use innovative concepts and treatment methods.”). In an additional fourth subscale, *therapeutic relationship* is assessed (8 items, e.g., “I am confident that my conceptualizations of client problems do not consist of stereotypes and biases.”). The MCI allows to compute both a total score and scores for the four subcategories. The 40 self-report items are answered on a 4-point Likert scale ranging from 1 (very inaccurate) to 4 (very accurate). Research indicates that the MCI is a suitable psychometrically robust instrument for evaluating transcultural competence in multicultural training processes [61]. The dimensions appeared to be distinct yet interrelated [61]. Internal consistency reliabilities (Cronbach’s alpha) ranged from .67 to .81 in the subscales and .86 for the full scale [44].

A second questionnaire on transcultural competencies is included, which was specifically developed for the German psychotherapeutic setting, the OnTracc-questionnaire [45]. It contains 28-self-report statements, which are based on three factors, *engagement* (7 items, e.g., “I am actively involved in the process of reducing barriers for refugees and people with a migration background in accessing psychotherapy (e.g. against the shortage of translators).”), *awareness* (10 items, e.g., “I take cultural related differences in symptom description into account.”), and *challenges* (11 items, e.g., “I worry about my skills to work with refugees, because they have a different understanding of diseases and mind.”). The items are rated on a 5-point Likert scale from 1 (totally disagree) to 5 (totally agree). The OnTracc-questionnaire showed substantial convergent and discriminant validity to the MCI subscales and good reliability for all subscales (McDonald’s omega between .73 and .81) [45]. The OnTracc-questionnaire was likewise recommended for evaluating transcultural competence in training [45].

#### Secondary outcome measures

In addition to transcultural competence, secondary outcomes serve to explore concrete experiences in treating patients with diverse cultural backgrounds. Eight items are used for this purpose. For example, the number of patients with diverse cultural backgrounds who are currently being treated by each participant in their clinical practice is recorded. The participants are further asked whether they are supervised in the field of transcultural therapy and whether they have ever worked with a language mediator. A 6-point Likert scale will additionally be used to assess satisfaction in therapies with patients with diverse cultural backgrounds and perceived competence. The items were taken from a preliminary study [62].

#### Satisfaction with training

In order to be able to adapt and improve the training, the training will be assessed in terms of satisfaction and acceptance. The questions were designed specifically for the study and adapted to the training content. They were tested and validated for comprehensibility during piloting. The questions refer to 5 sections: scope and practicability (5 items, e.g., “I was able to complete the training within the six weeks without any timing issues.”), formal design (5 items, e.g., “The design of the training platform was engaging.”), content (24 items, e.g., “The modules were varied.”), relevance to practice (4 items, e.g., “The training has made me feel more competent working with patients with diverse cultural backgrounds.”), and personal experience (8 items, e.g., “My expectations of the training were met.”). These questions are assessed on a 6-point Likert scale, from 1, strongly disagree, to 6, strongly agree. Participants further have the opportunity to write down any other comments and suggestions for improving the training in a free text field.

#### Control variables

In the pre-training assessment, sociodemographic data are collected through 18 items. In addition to standard socio-demographic information (age, gender, nationality), participants are asked whether they would perceive themselves as having a migration background. They are further asked whether they speak more than two languages fluently and whether they do/did any volunteer work for refugees or migrants.

In addition, questions are asked about therapeutic practice: duration of psychotherapeutic activity, specialization (behavioral therapy, depth psychology, etc.), and workplace (own practice, clinic, research institution, etc.). Finally, participants are asked whether, and if so to what extent, training with a focus on “culture in psychotherapy” has ever been completed.

### Participant timeline {13}

Via a link, participants can access the detailed description and the inclusion criteria of the study online. After accepting the informed consent, the participants will be forwarded to the pre-training assessment (t0). During the pre-training assessment, the control variables as well as the primary (Multicultural Counseling Inventory and OnTracc) and secondary outcomes are measured. The participants will subsequently be randomized. The TG will directly start with the training. After the training (t1) and 2 (t4) and 6 months later (t5), the assessments are carried out again. The WG will complete a control assessment (t1) after the training group has completed the training. They will also complete the 2-month follow-up assessment (t2). After the 2-month follow-up assessment, they will start the training. After the training (t3) and 2 (t4) and 6 months (t5) later, they will complete the assessments. For the participant timeline, please refer to Fig. 1.

### Sample size {14}

To the authors' knowledge, there are no web-based training programs for transcultural competence in Germany. For this reason, a concise estimate of the expected effect size is difficult to provide. A systematic review from 2020 on the effectiveness of cultural competence with nine studies showed effect sizes from small (*d* = 0.10) to large (*d* = 2.11) [63]. An online intervention to increase transcultural competence in the USA revealed medium effect sizes for the dimension attitudes and skills and large effect sizes for the dimension knowledge [40]. Based on the mixed results, we opted for a medium effect size.

A calculation with the Shiny App [64] with an effect size of *Glass’ delta* = 0.5 showed that a linear mixed model 3 (measurement time: pre, post, follow-up 1) × 2 (groups: training group vs. waitlist control group) requires a sample number of *N* = 174 (*α* = 0.05, power = 0.95).

### Recruitment {15}

Recruitment takes place via websites (e.g., state chamber of clinical psychologists), mailing lists (e.g., clinical psychologists, training institutes), advertisements (e.g., in German journals with a strong focus on therapeutic practice), and social media (therapist groups on Facebook or Instagram). Through a link, participants can obtain further information and access the study.

## Assignment of interventions: allocation

### Sequence generation {16a}

Participants will be randomly assigned to the training group or the waitlist control group. Blockwise randomization will be performed to make the groups equal in size until the target sample size is reached. The size of the blocks will remain hidden from the supervisors accompanying the training.

### Concealment mechanism {16b}

Implementation of the allocation sequence is allowed by Microsoft Excel [65]. The program randomly divides participants into evenly sized groups.

### Implementation {16c}

The randomization is performed by a research assistant who is not involved in the research project or in the training support. The research team of this study subsequently informs the participants of their condition by email.

## Assignment of interventions: blinding

### Who will be blinded {17a}

Due to the trial design, neither the participants nor the researchers will be blinded.

### Procedure for unblinding if needed {17b}

n/a. Since there is no blinding, no procedure for unblinding is required.

## Data collection and management

### Plans for assessment and collection of outcomes {18a}

All data will be collected online on the Unipark survey platform via participant self-report (https://www.unipark.com). The primary (transcultural competence) and secondary outcomes (experiences in treating patients with diverse cultural backgrounds) will be collected at each time point of measurement (pre, post, follow-up 1, follow-up 2). Control variables (sociodemographic information and therapeutic practice) will be assessed at baseline only. After training, satisfaction with the training (e.g., scope, practicability, formal design) will be assessed. For an overview of which questionnaires are applied at which time point, please refer to Fig. 1. For detailed information regarding the questionnaires, see {12}.

### Plans to promote participant retention and complete follow-up {18b}

Participant retention is promoted through weekly contact with a supervisor. For the follow-up surveys, participants are contacted by email. If the questionnaires are not completed within seven days, a reminder email will follow. If the questionnaire is still not answered, another e-mail will follow within a week. After 4 weeks, a last reminder to fill in the questionnaire follows. The emails emphasize the importance of completion in order to improve training and, in the long term, the availability of qualified training. At the same time, reference is made to the continuing education credit that participants receive after completing the final questionnaires. As all questionnaires can be answered online, participants can fill in the questionnaires flexibly and from any place. No further promotional measures will be taken to increase participation.

### Data management {19}

The data obtained in this study is collected and stored in compliance with the requirements of the General Data Protection Regulation of Germany (DSGVO). The data is collected using Unipark, a survey software that stores data encrypted on a German server; IP addresses are not stored in the log files. We will apply a key coding strategy for separating identified data from substantive data.

### Confidentiality {27}

The coding list is only accessible to the project leaders. It is stored on an encrypted local server at Philipps-Universität Marburg and deleted after completion of the data evaluation. The data will be treated strictly confidential.

### Plans for collection, laboratory evaluation, and storage of biological specimens for genetic or molecular analysis in this trial/future use {33}

n/a. No collection of biological specimens is required.

## Statistical methods

### Statistical methods for primary and secondary outcomes {20a}

Outcomes will be evaluated using intention-to-treat (ITT) analysis. Group differences (group: training group vs. waitlist control group) in primary (transcultural competence; MCI and OnTracc) and secondary outcomes (experiences in treating people from other cultures) will be examined using piecewise linear mixed-effects models with repeated measures (time point: pre, post, follow-up 1). Likely will piecewise linear mixed-effects models be used to examine the longer-term effect of the training (including the four time points: pre, post, follow-up 1, and follow-up 2). The significance level will be set at *p* < .05.

Sociodemographic data will be listed by group condition and for the total group. Group comparisons will be examined at baseline to check for successful randomization. Categorical data are reported as *N* (%), continuous data as mean (SD).

### Interim analyses {21b}

n/a. Neither interim analyses nor formal stopping rules will be applied in our study, as this is not a clinical trial and poses low safety concerns.

### Methods for additional analyses (e.g., subgroup analyses) {20b}

Regarding additional analyses, we would like to examine the clinical psychologist’s socio-demographic influence on the outcomes (e.g., age, duration of activity as a therapist, own migration background, multilingualism, volunteering for refugees and patients from different countries). We will investigate socio-demographic variables and disparities, including an examination of potential differences in the dependent variable among the three German-speaking countries. Furthermore, a consistency test will be performed to compare the post-intervention effects between the training group and the waitlist control group. For this purpose, two models are computed, one with the group as a predictor and one without, and the two models are compared with each other. We also would like to examine the influence of adherence on the outcomes by means of completed modules by a logistic regression.

### Methods in analysis to handle protocol non-adherence and any statistical methods to handle missing data {20c}

The data are analyzed according to the intention-to-treat principles, and data of all individuals randomized in this trial will be entered in our analyses. The handling of missing data is reported in tables and results.

### Plans to give access to the full protocol, participant-level data, and statistical code {31c}

The complete protocol of the presented study is in this document. On request, participant-level data, statistical code, or documentation can be provided by the authors.

## Oversight and monitoring

### Composition of the coordinating center and trial steering committee {5d}

The trial team consists of six researchers who exchange information on the progress of the study at two-week intervals. The principal investigator holds ultimate oversight of the study. The study coordinator is responsible for data collection and mentoring the supervisors. The supervisors are in charge of supervising the participants, i.e., they give feedback on the modules completed. The supervisors and the study coordinator will meet on a weekly basis to discuss upcoming issues concerning the supervision of participants. Due to the small project team, a Trial Steering Committee will not be constituted.

### Composition of the data monitoring committee, its role, and reporting structure {21a}

n/a. No extra monitoring committee is formed due to the fact the trial does not include a clinical sample and is a monocentric trial. The data monitoring is conducted by one of the investigators who is not directly involved in data collection.

### Adverse event reporting and harms {22}

n/a. Adverse event reporting and harms are not explicitly surveyed as the study does not include a clinical sample. Participants are informed that they can contact the study team, the study coordinator, or the principal investigators for questions or uncertainties.

### Frequency and plans for auditing trial conduct {23}

n/a. We will not install a Data Monitoring Committee for this trial, given that this is not a clinical trial involving a clinical sample, but an educational, low-risk intervention for clinical psychologists. The trial will be monitored by the principal investigator and three members of the research team. The supervisors and the study coordinator will meet on a weekly basis to discuss upcoming issues.

### Plans for communicating important protocol amendments to relevant parties (e.g., trial participants, ethical committees) {25}

Important protocol modifications (e.g., changes to eligibility criteria, outcomes) will be communicated to and must be approved by the local Ethics Committee. In addition, the protocol would be updated and the trial registry would be adjusted.

## Dissemination plans {31a}

The results will be published in peer-reviewed journals and presented at national and international conferences. Furthermore, participants will also be informed about the results. No publication restrictions are intended. If the study was successful in increasing transcultural competence, we will explore how to make the training available to clinical psychologists.

## Discussion

War, political and economic crises, natural catastrophes, and other threats of life and livelihood force people to leave their familiar environment. Forced migration is often accompanied by increased psychological distress and mental disorders [66]. Even when migration is deliberate, for example in the expectation of achieving a better standard of living, it is accompanied by psychological distress. Indeed, new challenges await in the country of arrival, often cited in research as post-migration living difficulties [66]. These include socio-economic difficulties (barriers to employment, housing, and financial insecurities), but also social and interpersonal challenges (social isolation, family separation, loss of social identity, discrimination). The asylum process (length of the asylum procedure, uncertain visa status) plays an important role, as does immigration policy in the host countries. These factors are further exacerbated by language barriers [66, 67]. For this reason, it is not surprising that an increased prevalence of mental disorders is often found in patients with diverse cultural backgrounds [6, 7]. Nevertheless, the recourse to psychotherapeutic treatment remains relatively limited [8].

There are objective barriers responsible for this condition, such as a lack of language mediators and difficulties with the financial takeover [9, 12, 18, 19]. But there are also barriers on the therapist’s side, such as shame and fear of increased effort (e.g., costs for translators) and stereotyping that prevent patient admission [13, 17, 24]. One way to improve care for patients with diverse cultural backgrounds runs through clinical psychologist training. The aim of such training is to promote transcultural competence and thereby reduce therapy dropouts and increase therapy success. If the study can demonstrate that transcultural competence can be fostered in clinical psychologists through our 6-week online training, the training could be used in the continuing education of clinical psychologists. The pandemic has highlighted how essential it is to offer flexible and time and place-independent training options that allow participants to complete the content at their own pace. Especially for people who work full time or are highly constrained by family responsibilities, online training enables a flexible opportunity for continuing education and eliminates travel and accommodation costs [68].

### Limitations

The following anticipated limitations of our trial will have to be considered for the interpretation of our findings: The control condition is a waitlist control group. A waitlist control group carries the risk of overestimating effects [69]. The design further does not enable blinding. The advantage of the WL is that it allows all participants to attend the training and to train their transcultural competence. Furthermore, there are no ethical concerns against a waiting period, since the content of the study is neither a clinical trial nor a clinical sample. Sima et al. [70] emphasized the importance of informing participants of the approximate start time in a waitlist control condition and not making them wait too long. Such an approach would lead to fewer dropouts. This remark can be taken into account in this trial.

Another limitation is that we rely on self-report assessments only to assess cultural competence, which is susceptible to various biases, including social desirability bias. It would be beneficial in future research to augment the self-report assessment by incorporating a performance test that exposes therapists to challenging transcultural therapy situations. These scenarios could be presented through video simulations, and clinical psychologists’ facilitative interpersonal skills could be evaluated [71, 72].

Further, participation requires an electronic device, Internet access, and basic computer skills. An attempt has been made to make the training as intuitive and user-friendly as possible. In the introduction of the training, there is information included on how to use the different functions (unfold texts, play videos). The feedback received from the feasibility study of the platform has enabled us to further simplify the navigation on the platform.

The training was created in German for the German-speaking countries. Conclusions about its effectiveness in other countries must be drawn with caution.

Finally, in a training of transcultural competence, the aspect of awareness, self-awareness, and self-reflection is essential [31]. This aspect is facilitated in exchange with other participants, which the online training does not allow. Nevertheless, we intended to create a sense of companionship with the three fictional therapists who also “attend” the training. These therapists repeatedly complement exercises with their own responses and self-reflections. Furthermore, the exchange with the accompanying supervisor remains possible at any time.

### Strengths

In Germany, there are only a few training that teach transcultural competence and examine them in a randomized controlled manner (for an example see [22]). To the authors' knowledge, no web-based training for teaching transcultural competence in Germany exists. The online format offers additional advantages: Participants can do the training in a time and location flexible way and independent of external influences (such as contact restrictions during a pandemic). The specific questionnaire after the training allows the training to be examined according to practicability, scope, and comprehensibility and to be adapted and improved in the long term. In addition, it is important for online training to receive the same accreditation as face-to-face training [68]. Our training has been accredited by the federal chamber of clinical psychologists, which is expected to lead to increased recognition. The implementation of the trial makes it possible to further improve and provide flexible continuing education for clinical psychologists.

## Trial status

The trial was registered on February 21, 2023, under https://www.bfarm.de/DE/Das-BfArM/Aufgaben/Deutsches-Register-Klinischer-Studien/_node.html, identifier DRKS00031105. Recruitment has started in April 2023 and is expected to be completed in April 2024. The protocol presented is the first version (July 04, 2023).

### Supplementary Information

**Additional file 1.**

## Abbreviations

WL: Waitlist control group
DSGVO: Datenschutz-Grundverordnung (General Data Protection Regulation)
MCI: Multicultural Counseling Inventory
TG: Training group

## Footnotes

1 In research, different terms are used, such as *people with a migration background*, *ethnic/racial minority groups*, *migrants*, *immigrants*, *asylum seekers*, and *refugees*. In this article, we consistently use the term *patients with diverse cultural backgrounds* if it concerns everyone and the specific one only if it concerns a study that explicitly addresses a group, e.g., asylum seekers.
